# Dietary Intake Trajectories from Early Life and Associated Health Outcomes: A Systematic Review

**DOI:** 10.1016/j.advnut.2025.100528

**Published:** 2025-09-29

**Authors:** Miaobing Zheng, Seon Y Park, Kristy A Bolton, Mary Foong-Fong Chong, Sara Grafenauer, Bo Xi

**Affiliations:** 1 School of Health Sciences, Faculty of Medicine and Health, University of New South Wales, Kensington, New South Wales, Australia; 2 Institute for Physical Activity and Nutrition, School of Exercise and Nutrition Sciences, Deakin University, Geelong, Australia; 3 Saw Swee Hock School of Public Health, National University of Singapore and National University Health System, Tahir Foundation Building, Singapore; 4 Institute for Human Development and Potential (IHDP), Agency for Science, Technology and Research (A∗STAR), Singapore; 5 Department of Epidemiology, School of Public Health, Qilu Hospital, Cheeloo College of Medicine, Shandong University/Children Cardiovascular Research Center of Shandong University, Jinan, China

**Keywords:** Dietary trajectories, trajectory modeling, longitudinal, early life, health outcomes

## Abstract

The vital role of early dietary intake in shaping later health has been widely acknowledged, but how changes in dietary intake from early life influence health outcomes remains unclear. This review systematically synthesized the literature that examined the longitudinal associations between changes in dietary intake (i.e., trajectories) from early life and health outcomes. Electronic searches were conducted in PubMed, Embase, and ScienceDirect to gather longitudinal cohort studies that investigated dietary intake trajectories from infancy, childhood, or adolescence (with first dietary assessment before age 18 y) and any health outcomes published from inception to September 2024 (CRD42024512716). Of 16 included studies, 14 reported significant associations between dietary intake trajectories (intakes of macronutrients, food groups, diet quality or dietary patterns) in childhood or from childhood to adolescence/early adulthood and dental, obesity, cardiometabolic, neurocognitive, liver or gut health outcomes from age 2 y up to age 41 y. Most studies were of high (*n* = 2) or acceptable (*n* = 12) quality. A high or increasing sugar intake trajectory during infancy was linked to an increased risk of dental caries in early childhood, whereas a healthy dietary pattern trajectory was associated with lower risk. Trajectories of poor diet quality, red meat dietary pattern, and high discretionary food intake from infancy/early childhood were associated with adiposity and adverse cardiometabolic outcomes at adolescence and adulthood. Significant associations were also found between trajectories of dietary patterns or macronutrient intakes (e.g., protein, carbohydrate, and dietary fiber) from infancy and neurocognitive outcomes in childhood. High energy intake trajectory from early childhood to adolescence and carbohydrate intake from infancy to early adulthood were associated with poor liver health outcomes and gut microbiota composition in adulthood, respectively. Dietary intake trajectories established from infancy or early childhood were associated with various health outcomes. Dietary interventions should be initiated from infancy or early childhood for early health promotion.

**Statement of Significance**

To our knowledge, this is the first review to systematically synthesize the literature that examined the longitudinal associations between changes in dietary intake (i.e., trajectories) from early life and health outcomes.

## Introduction

Childhood is a critical period for the development of health behaviors and the programming of later health [1]. Nutrition in early life has been associated with various subsequent health outcomes later in life. Unhealthy diets with poor diet quality during childhood, characterized by excess consumption of energy, saturated fats, added sugars, and discretionary foods, have been linked with adverse health outcomes such as dental caries, obesity, or cardiovascular disease [2]. In contrast, healthy diets with good diet quality including high consumption of wholegrains, fruits and vegetables, healthy protein, fats, and essential nutrients in childhood, are associated with favorable health outcomes later in life [2].

The existing evidence, however, largely comes from prospective cohort studies where associations between dietary intakes at a single time point and health outcomes at 1 later time point were examined [3,4], neglecting the potential changes in dietary intake over time. Emerging studies utilized longitudinal trajectory modeling to explore changes in dietary intake over time (i.e., trajectories) and assessed their associations with later health outcomes [[5], [6], [7], [8], [9]]. Assessing the longitudinal associations between dietary intake trajectories and later health outcomes can provide a deeper understanding of the dynamic association over time and identify critical time points when the association starts to emerge or is most significant [10,11]. Longitudinal cohort studies also provide stronger evidence than cross-sectional studies by offering valuable insights on temporal order of the relationship.

Previous systematic reviews have summarized evidence from the longitudinal studies that investigated the tracking of dietary intake [12,13] or changes in dietary intake from adolescence into early adulthood [14]. Tracking of dietary intake is evaluated by assessing the correlation of dietary intake between 2 time points. In contrast to longitudinal trajectory modeling, tracking does not capture direction or magnitudes of change in dietary intake over time. Given evidence has shown that food preferences and dietary habits are established early in life [15], it is crucial to examine the impact of dietary intake trajectories from early life on later health. A recently published review in 2022 summarized the literature on dietary intake trajectories across the life span from early life to adulthood, but it largely focused on summarizing findings on critical timing of dietary change across the lifespan and associated factors, and it did not utilize a systematic approach [16]. To date, no systematic review has summarized the existing studies examining the longitudinal associations between dietary intake trajectories from early life (i.e., infancy) and health outcomes. Therefore, this systematic review aimed to summarize findings from longitudinal studies that investigated dietary intake trajectories from early life and their associations with health outcomes. It aims to shed light on the various aspects of dietary intakes (e.g., intakes of nutrients, food groups, dietary quality, or patterns) that have been examined as trajectories in early life as well as the range of health outcomes investigated.

## Methods

### Eligibility criteria

The review included longitudinal cohort studies that have repeated measurements of dietary intake over 3 or more time points and assessed the association with any health outcomes. Eligible studies may involve any dietary variables such as energy or nutrient intakes, food group intakes, diet quality scores, dietary patterns, but require having 3 dietary assessments with the first dietary measurement taken any time from infancy to adolescence (from birth to age 18 y). Studies were excluded if they focused on appetite or eating behaviors, involved only adults or individuals with special diseases, or concentrated on association between maternal diet and child health. Only studies published in English and conducted with healthy human subjects were included, with no exclusions on body weight or the age at final follow-up. The systematic review adhered to PRISMA guidelines, and its protocol was registered at PROSPERO (CRD42024512716).

### Search strategy

Literature searches were conducted in PubMed, Embase, and ScienceDirect, using 3 domains of key terms related to “dietary intake” (“dietary intake” OR “diet” OR “nutrient intake” OR “nutrient∗” OR “food∗” OR “food intake∗” OR “dietary pattern∗” OR “dietary quality” OR “diet quality” OR “nutrition” OR “micronutrient∗” OR “macronutrient∗”), “trajectories” (“trajectories” OR “trajectory” OR “changes” OR “change”), and “childhood” (“child∗” OR “adolesce∗” or “pediatric” OR “paediatric” OR “youth” OR “boy∗” OR “girl∗” OR “students”). The search covered articles published from inception to September 2024. Additionally, a manual search of Google Scholar and the reference lists of relevant articles was performed to identify additional eligible studies.

### Study selection and extraction

Articles were imported into Endnote X9 (Clarivate), and duplicates were removed. A 2-step screening process was employed, initially screening titles and/or abstracts, followed by full-text review. MZ and SYP independently conducted the initial screening and full-text review, with conflicts resolved through discussion with the third reviewer (KAB). Studies meeting eligibility criteria underwent data extraction, including details such as sample characteristics (i.e., study country, sample size, cohort name, and children's ages), dietary intake variables, dietary assessment methods, ages of dietary assessment, trajectory modeling methods, the number of identified trajectories, health outcomes, timing of health outcome assessment, adjusted confounders, and study findings. Data extraction was conducted independently and in duplicate by 3 reviewers (SYP, KAB, and MZ), with any discrepancies resolved through consensus discussion.

### Risk of bias assessment

Quality assessment was performed independently by 2 reviewers (SYP and MZ) using the Scottish Intercollegiate Guidelines Network 50 methodology checklist for cohort studies [17]. The checklist consisted of 16 items focusing on 5 essential criteria [18]: subject comparability, reliability of exposure assessment, validity of outcome measures, reliability of repeated exposure measures, and reporting of confidence intervals (CIs). Studies meeting all 5 criteria were rated as high quality with minimal risk of bias, those meeting 3–4 criteria were rated as acceptable, and those meeting 1–2 criteria were considered low quality.

## Results

### Study Selection

**FIGURE 1:**
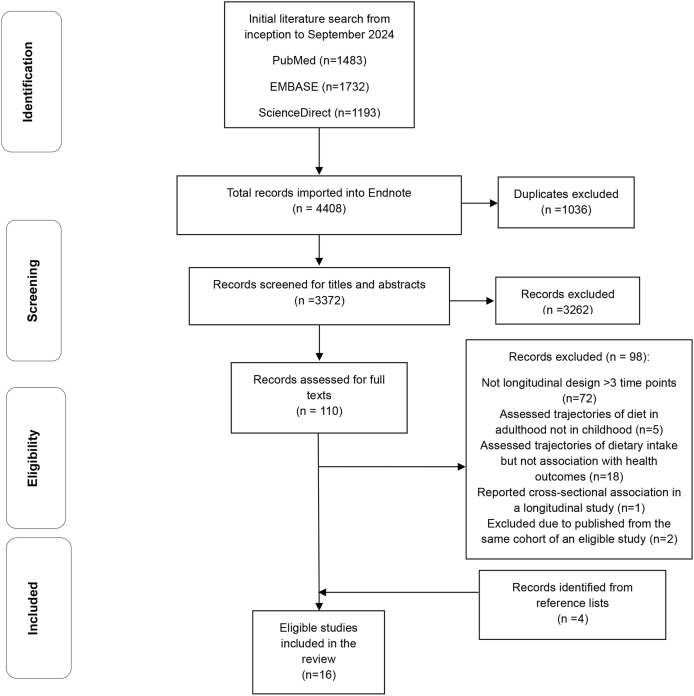
Flow chart for selection of studies examining the association between dietary intake trajectories from early life and health outcomes.

A total of 4408 citations were retrieved from 3 databases and other sources. After removing 1036 duplicates, 3372 citations underwent initial screening of titles and/or abstracts using Covidence. Following this, 110 studies were selected for full-text review. Of these, 72 studies without dietary intake assessment over 3 time points or more were excluded. For example, a study defined vitamin D trajectory groups based on baseline and follow-up data only were excluded [19]. Another study assessed the associations between sugary food intake at 2 time points in toddlerhood with neurocognitive development in early childhood was excluded [20]. Five studies assessed dietary intake trajectories in adulthood only were also excluded [[21], [22], [23], [24], [25]]. Eighteen studies assessed factors associated with trajectories or tracking of dietary intake without evaluating their associations with health outcomes were further excluded. Despite using longitudinal data, 1 study reported cross-sectional associations between food group intakes (water, fruit, vegetable, food, and drinks with added sugar) and BMI at ages 6, 11, and 15 y was also excluded [26]. Three studies reported an association between diet quality trajectories and adiposity outcomes from the Southampton Women's Survey [[27], [28], [29]]. The study reported an association between dietary trajectories and health outcome at a later follow-up was included in the current review [28]. Moreover, the included study assessed dietary intakes of both mothers and their children, only child data were extracted for the current review [28]. Four additional eligible articles were identified from reference lists and included for subsequent data extraction. The total number of eligible studies for inclusion was 16 (Figure 1).

### Study characteristics

The 16 included longitudinal cohort studies reported findings from 13 cohorts with sample sizes ranging from 128 [30] to 7652 participants [31] (Table 1) [[6], [7], [8], [9],^28^,[30], [31], [32], [33], [34], [35], [36], [37], [38], [39], [40]]. Cohorts were predominantly from Asia Pacific nations, namely Australia (*n* = 7) [[6], [7], [8], [9],[37], [38], [39]], Singapore (*n =* 2) [34,40], Philippine (*n =* 1) [33], followed by Europe with studies from the United Kingdom (*n =* 3) [28,31,32], Germany (*n =* 1) [30], Finland (*n =* 1) [35], as well as with 1 study from Brazil (*n =* 1) in South America [36]. Of 16 studies, 8 studies [6,8,31,32,34,[38], [39], [40]] utilized data from 4 cohorts, but they were considered separately in the current review as associations between different dietary intake trajectories and different health outcomes at different follow-up time points were assessed.

**TABLE 1:** Longitudinal studies assessing the association between dietary intake trajectories from early life and health outcomes.

Author, year	Country, sample size, cohort name	Dietary variable	Dietary assessment method	Ages at dietary assessment (number of data points)	Trajectory modeling methods	Identified dietary trajectories	Health outcomes	Age at outcome assessment	Adjusted confounders	Main findings	Study quality
Smithers et al., 2013 [31]	United Kingdom, *n =* 7652, ALSPAC	Dietary pattern: Healthy Discretionary Traditional Ready-to-eat	FFQ	6, 15, 24 m (3)	Multilevel mixed models	Sample average trajectory (Intercept at 6 m and slope from 6 to 24 m) Slope: rate of change	Neurocognitive outcomes intelligence quotient (IQ) (measured)	8, 15 y	Child sex, gestational age, birth weight, ethnicity, singleton/twin, maternal age, parity, social class, education, family income, maternal smoking, home environment	IQ at 8 y Healthy pattern intercept NS; slope + sig (β: 1.07; 95% CI: 0.17, 1.97) Discretionary pattern (intercept/slope) NS Traditional pattern (intercept/slope) NS Ready to eat pattern (intercept/slope) NS IQ at 15 y Healthy pattern intercept +sig (β 0.75; 95% CI 0.00, 1.50); slope NS Discretionary pattern intercept NS; slope -sig (β: –0.73; 95% CI: –1.33, –0.14) Traditional pattern intercept NS; slope -sig (β: –0.41; 95% CI: –0.77, –0.04) Ready to eat pattern (intercept/slope) NS	Acceptable
Anderson et al., 2015 [32]	United Kingdom, *n =* 3188, ALSPAC	Energy and nutrient intakes: energy, carbohydrate, sugar, starch, fat, fat subtypes intakes	FFQ at 3,7 y and 3-d food diaries at 13 y	3, 7, 13 y (3)	Linear spline multilevel models	Trajectories of energy and macronutrient intakes	Liver outcomes Ultrasound scan (USS)-measured liver fat, USS-measured liver stiffness, ALT, AST, GGT (measured)	18 y	Child sex, age at outcome assessment, maternal pre-pregnancy BMI, maternal age, social class, maternal education, and parity	Energy intakes at 3 y liver fat at 18 y: + sig (OR: 1.79; 95% CI: 1.14, 2.79) ALT at 18 y: + sig (OR: 7.00; 95% CI: 4.00, 10.00) GGT at 18 y: + sig (OR: 5.00; 95% CI: 2.00, 7.00) Liver stiffness/AST: NS Energy intake at 7 y liver fat at 18 y: +sig (OR: 1.30; 95% CI: 1.06, 1.60) ALT at 18 y: + sig (OR: 3.00; 95% CI: 2.00, 5.00) GGT at 18 y: +sig (OR: 2.00; 95% CI: 0.00, 3.00) Liver stiffness/AST: NS Energy intake at 13 y ALT at 18 y: +sig (OR: 4.00; 95% CI: 2.00, 5.00) AST at 18 y: +sig (OR: 1.00; 95% CI: 0.00, 2.00) GGT at 18 y: +sig (OR:3.00; 95% CI: 0.00, 4.00) Liver fat/stiffness: NS Positive associations were found between macronutrient intakes and some liver outcomes, but there was no consistent pattern. Most associations attenuated to null after energy intake adjustment.	Acceptable
Wright et al., 2017 [33]	Philippines, *n =* 2586, CLHNS study	Energy and nutrient intakes: protein intakes	1-d 24-h recall at 2 y, 2-d 24-h recalls from 11 y onwards	2, 11, 15, 19, 22 y (5)	Latent growth curve modeling	4 trajectory groups: Normal consumers (58%), High consumers (20%), Usually high consumers (18%), Always high consumers (5%)	Overweight/obesity BMI, lean mass, and fat mass	22 y	Offspring birth weight, maternal education, maternal height at birth; offspring BMI and household assets at age 2 y; offspring education and assets, physical activity level, carbohydrate/fat residuals, and energy intakes at age 22 y	Usually high protein vs. normal consumers BMI at 22 y: -sig in males: (β: –0.244; 95% CI: –0.406, –0.083) in females: (β: –0.338; 95% CI: –0.553, –0.122) Fat mass at 22 y: -sig in males: (β: –0.170; 95% CI: –0.329, –0.011), in females: (β: –0.374; 95% CI: –0.594, –0.155) Lean mass at 22 y: -sig in males: (β: –0.388; 95% CI: –0.541, –0.236), in females: (β: –0.296; 95% CI: –0.506, –0.086) Always high protein vs. normal consumers Lean mass at 22 y: -sig In male: (β: –0.441; 95% CI: –0.781, –0.101), In female: NS High vs. normal consumers: NS Protein intake at 2 y and BMI/lean mass at 22 y in females: +sig Protein intake at other ages and BMI/lean mass/fat mass at 22 y in males/females: -sig	Acceptable
Kerr et al., 2018 [7]	Australia, *n =* 188, PEAS study	Diet quality score (0–14 scores)	4-d food diaries	4, 4.5, 5, 5.5, 6, 6.5, 10, 15 y (8)	Latent class trajectory modeling	4 trajectory groups: Healthy (21%) Moderately healthy (46%) Moderately Unhealthy (25%) Unhealthy (8%)	Cardiometabolic makers resting heart rate, blood pressure, pulse wave velocity, carotid intima-media thickness, retinal arterio-to-venule ratio (measured)	15 y	Maternal education, SEIFA at 4 y, Child: age, sex, physical activity, puberty, BMI z-score at 15 y	Unhealthy vs. healthy trajectories Resting heart rate at 15 y: + sig (β: 10.50; 95% CI: 2.90, 18.00) Moderately unhealthy vs. healthy trajectories Resting heart rate at 15 y: NS (β: 4.50; 95% CI: –0.70, 9.70) Moderately healthy vs. healthy trajectories Resting heart rate at 15 y: NS (β: 4.10; 95% CI: –0.60, 8.90) Diet quality trajectories and other cardiovascular outcomes: NS	Acceptable
Hu et al, 2019 [34]	Singapore, *n =* 363, GUSTO study	Dietary pattern: predominantly breastmilk, guidelines, easy-to-prepare foods, noodles (in soup) and seafood	3-d food diaries	6,9,12 m (3)	Multilevel mixed models	Sample average trajectory (Intercept at 6 m and slope from 6–12 m) Slope: rate of change	Dental caries -caries present/absent, decayed teeth, decayed surfaces (measured)	2–3 y	Sociodemographic characteristics, oral hygiene habits, perinatal and postnatal characteristics	Decayed surface at 2–3 y: Guidelines dietary pattern at 6 mo (intercept): - sig (adjusted-incidence risk ratio (IRR): 0.26; 95% CI: 0.12, 0.53) Guidelines dietary pattern from 6 to 12 mo (slope): -sig (IRR: 2.4 × 10^-4^; 95% CI: 4.2 × 10^-7^, 0.13) Other dietary patterns (intercept/slope) and dental surfaces: NS	Acceptable
Kerr et al., 2021 [8]	Australia, *n =* 1861, LSAC	Diet quality score (0 -14 scores)	FFQ	2–3 y, 4-5 y, 6-7 y, 8-9 y, 10-11 y (5)	Group-based trajectory modeling	4 trajectory groups: always healthy (56%); moderately healthy (21%); becoming less healthy (17%); never healthy (7%)	Cardiometabolic makers: resting heart rate, systolic/diastolic blood pressure, pulse wave velocity, carotid elasticity/distensibility, structural phenotypes (carotid intima-media thickness, retinal microvasculature) Metabolic risk score (measured)	11-12 y	Age, sex, family-level socioeconomic position	Becoming less healthy vs. always healthy Diastolic blood pressure: +sig (β: 1.00; 95% CI: 0.10, 1.90) Never healthy vs. always healthy Resting heart rate: + sig (β: 2.60; 95% CI: 0.40, 4.70) Metabolic risk score: +sig (β: 0.23; 95% CI 0.01, 0.45) Carotid elasticity: -sig (β: –0.30; 95% CI: –0.60, –0.10) Carotid distensibility: - sig (β: –1.20; 95% CI: –1.90, –0.50) Heart rate, distensibility, and diastolic blood pressure was progressively poorer for less healthy diet trajectories (linear trends p ≤ 0.02). Systolic blood pressure, pulse wave velocity, and structural phenotypes: NS	Acceptable
Manohar et al., 2021 [9]	Australia, *n =* 738, HSHK study	Food group intakes: core foods, discretionary, sugary foods	FFQ	4 m, 8 m, 1 y,2 y,3 y (5)	Group-based trajectory modeling	3 trajectory groups. Core foods Lowest (22.9%) Medium (43.6%), Highest (33.5%) Discretionary foods Lowest (40.8%) Medium (44.8%) Highest (14.4%) Sugary foods Lowest (41.3%) Medium (45.3%) Highest (13.4%)	Overweight/obesity and dental caries (measured)	3–4 y	Child age, gender, maternal age, marital status, number of children, maternal education, working status, index of socioeconomic advantage, infant feeding, country of smoking practices during pregnancy	Discretionary foods Highest vs. lowest group overweight/obesity risk: +sig (OR: 2.51; 95% CI: 1.16, 5.42) Medium vs. lowest: NS Core food intake trajectories and overweight/obesity risk: NS Sugary food intake trajectories and dental caries: NS	Acceptable
Oluwagbemigun et al., 2021 [30]	Germany, *n =* 128, DONALD study	Energy and nutrient intakes: energy, carbohydrate, fiber, protein, and fat	3-d weighted food records	Annually from 1 y to 18 y (18)	Multilevel mixed models	Sample average trajectory (Intercept at 1 y and slope from 1 to 18 y) Slope: rate of change	Gut microbiota composition (measured)	18 y	Child sex, sibling, birth weight and length, maternal BMI, maternal gestational weight, maternal education/ occupation, cesarean delivery, birth order, smoking, alcohol consumption, smoking, physical activity, BMI trajectory, child age at fecal sampling	Carbohydrate intakes at 1 y (intercept) and *Phascolarctobacterium*: - sig (β: –4.31, false discovery rate (FDR)∗-adjusted *P* = 0.006) Carbohydrate intakes at 1 y (intercept) and *Dialister*: +sig (β: 3.06, FDR-adjusted *P* 0.003) The carbohydrate intake from 1–18 y (slope) and *Desulfovibrio*: +sig (β: 13.16, FDR-adjusted *P* < 0.001) Energy/other macronutrients and gut microbiota composition: NS	Acceptable
Wu et al., 2021 [35]	Finland, *n =* 980, YFS study	Dietary pattern: traditional Finnish, High-carbohydrate foods, vegetable and dairy products, traditional Finish and high carbohydrate, red meat, Healthy	2-d 24-h recalls at first 3 time points plus FFQ at the remaining 2 time points	5 time points from age 3–18 y with 31 y follow-up until mean age of 41 y (5)	Group-based trajectory modeling	3 trajectory groups were identified for each dietary pattern	Cardiometabolic makers - impaired fasting glucose (IFG) (measured)	41 y	Child age, sex, BMI, serum 25-hydroxyvitamin D concentrations, total energy intake, parental history of diabetes, physical activity, smoking, socioeconomic status at baseline, and adult BMI	Trajectories of increased or stably medium adherence to the red meat pattern from youth to adulthood and IFG at 41 y: + sig (relative risk (RR): 1.46; 95% CI: 1.12, 1.90) For other dietary patterns and IFG: NS	Acceptable
Dalrymple et al., 2022 [28]	United Kingdom, *n =* 2936, SWS	Diet quality score (calculated from principal component coefficients of 49 food groups)	FFQ	6 m, 12 m, 3 y, 6–7 y, 8–9 y (5)	Latent class trajectory modeling	5 trajectory groups: Poor (5%), poor-medium (23%), Medium (39%), medium-better (28%), Best (5%).	Overweight/obesity BMI *z*-scores, arm/waist circumference, dual-energy X-ray absorptiometry (DXA) total body fat, % body fat, total lean mass, %lean mass (measured)	8–9 y	Maternal prepregnancy BMI, maternal highest education attainment, maternal age at birth and parity, child sex and age at the 8–9-y visit	One group/class decrease (poorer) in diet quality trajectory Higher BMI *z*-score at 8–9 y: +sig (βtrend: 0.08 95% CI: 0.00, 0.16) Higher percentage body fat at 8–9 y: + sig (βtrend: 0.08; 95% CI: 0.01, 0.15) Lower percentage lean mass at 8–9 y: -sig (βtrend: –0.08; 95% CI: –0.14, –0.01) Diet quality trajectories and other outcomes: NS	Acceptable
Echeverria et al., 2022 [36]	Brazil, *n =* 2806, 2015 Pelotas Birth Cohort	Food groups: sugar consumption score	Simple binary questions (yes vs. no)	3, 12, 24, 48 m (4)	Group-based trajectory modeling	4 trajectory groups: Always low (22.1%), Always intermediate (44.0%), Increasing (27.6%), Always high (6.3%)	Dental caries (dental caries experience/cavitated dental caries) (measured)	48 m	Family income, maternal education, maternal age, mother oral health instruction	Always intermediate vs. always low Dental caries experience: +sig (PR: prevalence ratio): 1.21; 95% CI: 1.03, 1.41 Cavitated dental caries: +sig (PR: 1.22; 95% CI: 1.00, 1.48) Increasing vs. always low Dental caries experience: +sig (PR: 1.50; 95% CI: 1.29, 1.74) Cavitated dental caries: +sig (PR: 1.60; 95% CI: 1.33, 1.92) Always high vs. always low Dental caries experience: +sig (PR:1.42; 95% CI: 1.17, 1.73) Cavitated dental caries: +sig (PR: 1.51; 95% CI: 1.19, 1.92)	Low
Cosier et al., 2023 [6]	Australia, *n =* 4360, LSAC	Diet quality score (0–14 scores)	FFQ	4–5 y, 6–7 y, 8–9 y, 10–11 y (4)	Group-based trajectory modeling	4 trajectory groups: Never healthy (4.3%); moderately healthy (23.1%); becoming less healthy (14.2%); always healthy (58.4%)	Cardiometabolic makers [blood pressure: systolic blood pressure (SBP), diastolic blood pressure (DBP)] (measured)	10–11 y	Child age, sex, indigenous status, SEP, maternal education and country of birth (model 1), maternal and paternal BMI and maternal hypertension in pregnancy (model 2), breastfeeding, pubertal status and physical activity (model 3), child BMI (model 4); child fat mass index (model 5)	Becoming healthy vs. never healthy SBP: -sig (model 3) (β: –2.19; 95% CI: –3.93, –0.45) Always healthy vs. never healthy SBP: -sig (model 3) (β: –2.19; 95% CI: –3.78, –0.59) Becoming healthy vs. never healthy DBP: -sig (model 3) (β: –1.54; 95% CI: –2.89, –0.20) Always healthy vs. never healthy DBP: -sig (model 3) (β: –1.71; 95% CI: –2.95, –0.47) Always healthy vs. never healthy DBP: -sig (model 4) (β: –1.34; 95% CI: –2.54, –0.14) Always healthy vs. never healthy DBP: -sig (model 5) (β: –1.23; 95% CI: –2.43, –0.03) Other trajectories and SBP/DBP: NS	Acceptable
Ha et al., 2023 [37]	Australia, *n =* 879, SMILE study	Energy and nutrient intakes: free sugar intakes in grams	1-d 24-h recall plus 2-d food diaries at 1 y, FFQ at 2, 5 y	1, 2, 5 y (3)	Group-based trajectory modeling	3 trajectory groups: high and increasing (16.6%); moderate and increasing (68.3%); Low and increasing (15.1%)	Dental caries Prevalence, caries experience (measured)	5 y	Child age, sex, birth weight, breastfeeding, maternal education, index of social disadvantage, household income, maternal country of birth, household composition	High and increasing vs. low and increasing prevalence of dental caries: +sig (prevalence ratio: 2.13; 95% CI: 1.23, 3.70) High and increasing vs. low and increasing caries experience: + sig (rate ratio: 2.10; 95% CI: 1.30, 3.30) For moderate and increasing trajectory and dental outcomes: NS	Low
Park et al., 2023 [38]	Australia, *n =* 328, INFANT study	Diet quality score (0 –9 scores) Breakfast quality index (BQI)	3-d 24-h recalls	1.5, 3.5, 5 y (3)	Group-based trajectory modeling	2 trajectory groups: High (74%); Low (26%)	Overweight/obesity (BMI z-score, child overweight status) (measured)	5 y	Child age, sex, birth weight Child sex, maternal country of birth, maternal education, prepregnancy BMI, child total energy intake at 1.5 y (model 1), breastfeeding duration (model 2)	Low BQI trajectory vs. high BQI Overweight risk: NS (OR: 1.31; 95% CI: 0.54, 3.19) (model 2) BMI *z*-score: NS (β: –0.01; 95% CI: –0.21, 0.19) (model 2)	High
Thorsteinsdottir et al., 2023 [39]	Australia, *n =* 503, INFANT study	Energy and nutrient intakes: fiber intakes in grams	3-d 24-h recalls	9, 18, 42, 60 m (4)	Group-based trajectory modeling	4 trajectory groups: Low (52.3%), Moderate (32.2%), High (13.3%), Unstable (2.2%)	Overweight/obesity (BMI z-score, child overweight status) (measured)	60 m	BMI *z*-score at 9 mo (model 1), treatment group, child sex, breastfeeding duration, the timing of solid food introduction, maternal employment status, education, country of birth and prepregnancy BMI (model 2), total energy intake (model 3)	Low vs. high and unstable fiber intake BMI z-score: NS (β: –0.12; 95% CI: –0.31, –0.07) (model 3) Child overweight status: NS (OR: 0.94; 95% CI: 0.51, 1.73) (model 3)	High
Toh et al., 2023 [40]	Singapore, *n =* 1247, GUSTO study	Energy and nutrient intakes: energy, protein, total fat, carbohydrate, dietary fiber	1-d food diary	6, 9, 12 m (3)	Multilevel mixed models	Sample average trajectory (Intercept at 6 m and slope from 6–12 m) Slope: rate of change	Neurocognitive outcomes (BSID-III test: cognition, expressive language, receptive language, fine motor and gross motor, etc.)	24, 54 m	Child age, maternal ethnicity, maternal education level, breastfeeding duration, child gestational age, child sex, and childbirth weight, child age at neurodevelopment testing	Protein intake at 6 m (intercept) Fine motor score at 24 m: +sig (β: 0.17; 95% CI: 0.03, 0.31) Protein intake from 6 to 12 m (slope) Fine motor at 24 m: +sig (β: 0.62; 95% CI: 0.10, 1.14) Total fat intake at 6 m (intercept) Receptive language score at 24 m: +sig (β: 0.04; 95% CI: 0.003, 0.07) Total fat intake from 6 to12 m (slope) Expressive language at 24 m: -sig (β: –0.20; 95% CI: –0.39, –0.01) Fine motor score at 24 m: - sig (β: –0.29; 95% CI: –0.48, –0.10) Carbohydrate intake at 6 m (intercept) Gross motor score at 24 m: -sig (β: –0.07; 95% CI: –0.14, –0.005) Carbohydrate intake from 6–12 m (slope) Receptive language at 24 m: +sig (β: 0.44; 95% CI: 0.08, 0.81) Fine motor scores at 24 m: +sig (β: 0.56; 95% CI: 0.18, 0.93) Fiber intakes from 6–12 m (slope) Fine motor scores at 24 m: +sig (β: 0.63; 95% CI: 0.16, 1.10) Nutrient intake trajectories and cognition and gross motor at 24 m: NS Nutrient intake trajectories and outcomes at 54 m: NS	Acceptable

Abbreviations: ALT, Alanine aminotransferase; AST, aspartate aminotransferase; β, beta-coefficient, CI, confidence interval; FFQ, food frequency questionnaire; GGT, gamma-glutamyl transferase, OR, odds ratio; +significant: positive significant association (*P* < 0.05); -significant: negative significant association (*P* < 0.05); NS, no significant association; ND, not described. BSID-III: Bayley Scales of Infant and Toddler Development, Third EditionDONALD: Dortmund Nutritional and Anthropometric Longitudinally Designed StudyALSPAC: Avon Longitudinal Study of Parents and ChildrenSEIFA: Socio-Economic Indexes for AreasINFANT: Infant Feeding, Activity and Nutrition TrialCLHNS: Cebu Longitudinal Health and Nutrition SurveyLSAC: Longitudinal Study of Australian ChildrenHSHK: Healthy Smiles Healthy KidsYFS: Cardiovascular Risk in Young Finns StudySWS: Southampton Women’s SurveySMILE: Study of Mothers' and Infants' Life Events Affecting Oral HealthGUSTO: Growing Up in Singapore Towards healthy Outcomes

In terms of dietary assessment methods, 5 of 16 studies used 3-d 24 h recalls [38,39], 3-d weighed food records [30], or 3–4 d food diaries [7,34]. Of 5 studies using food frequency questionnaires (FFQs) [6,8,9,28,31], only 1 study used validated FFQs [28]. A further 3 studies utilized 1- or 2-d dietary recalls [33,40] or food group-specific binary dietary questions with yes or no responses [36]. Three remaining studies used different dietary assessment methods (1–3 d 24 h recalls or food diaries, FFQ) across time points [32,35,37].

The baseline and final ages of dietary assessment ranged from 3 mo to 4–5 y and from 1 y to 22 y, respectively. Of 16 studies, 9 studies assessed changes in dietary intake in childhood (≤9 y) [9,28,31,34,36,37,[38], [39], [40]] and 7 studies examined changes in dietary intake from childhood to adolescence or early adulthood (10–22 y) [[6], [7], [8],^30^,^32^,^33^,^35^]. The number of repeated measurement time points for dietary assessment ranged from 3 to 18 [30].

Eleven of 16 studies utilized group-based trajectory modeling (GBTM) [6,8,9,35,36,38,39] or latent class trajectory modeling (LCTM) [7,28,33,37], which identified subgroup within a population that followed distinct dietary intake trajectories. Five studies employed multilevel mixed models or linear spline multilevel models [30,31,32,34,40], from which average dietary intake trajectory of the study sample was constructed.

Regarding the assessed dietary intake trajectories, various dietary intake trajectories were examined, including intake of energy or nutrient intakes, intakes of food groups, dietary quality, and dietary patterns. Six studies examined energy and/or nutrient intake trajectories [30,32,33,37,39,40]: intakes of total energy and macronutrients (i.e., carbohydrate, protein, and fat) (*n =* 3) [30,32,40], protein intake (*n =* 1) [33], free sugar intake (*n =* 1) [37], and dietary fiber intake (*n =* 1) [39]. Only 2 studies assessed trajectories of food group intakes (i.e., core, discretionary, and sugary foods or drinks) [9,36]. Five studies analyzed dietary quality trajectories [[6], [7], [8],^28^,^38^]. Three of these studies utilized the same dietary quality index, which included food groups and nutrients such as fruit, vegetables, water, absence of fatty foods, sugary foods, and sweetened drinks, ranging from 0 to 14 scores [[6], [7], [8]]. The other study investigated the "healthy" diet quality index derived using principal component analysis [28]. The remaining study evaluated the nutritional quality of breakfast with scores ranging from 0 to 9 derived from 9 items including breakfast cereal, wholegrains, dairy products, fruit, vegetables, calcium, energy, absence of added sugar, and butter/margarine [38]. Higher scores indicate better diet or breakfast quality. For the 3 studies examining dietary pattern trajectories [31,34,35], trajectories of 4–6 dietary patterns were assessed. For example, 2 studies examined 4 dietary patterns of “Healthy,” “Discretionary,” “Traditional” and “Ready to eat” [31] or “predominantly breastmilk”, “guidelines,” “easy-to-prepare,” and “noodle and seafood patterns,” respectively [34]. The remaining study evaluated 6 dietary patterns including “traditional Finnish pattern,” “high carbohydrate,” “vegetable and dairy products,” “traditional Finnish and high carbohydrate,” “red meat,” and “healthy” [35]. Detailed dietary intake variables for each study are presented in Supplemental Table 1.

Regarding health outcomes, 3 of 16 studies examined dental outcomes [34,36,37]. Four studies assessed adiposity outcomes (e.g., overweight status, BMI *z*-scores, or body composition) [28,33,38,39]. A further study investigated both childhood overweight and dental outcomes [9]. There were 4 studies exploring cardiometabolic outcomes such as resting heart rate, blood pressure, impaired fasting glucose, and hyperglycemia [[6], [7], [8],^35^]. Other health outcomes being assessed included neurocognitive outcomes (i.e., intelligence quotient [31], fine/gross motor or expressive/receptive language scores) [40], liver health markers [32], and gut microbiota composition [30]. Final age at health outcome assessments spanned from 2 y [34] to 41 y [35].

Commonly adjusted confounders across studies included child sex, gestational age, birth weight, ethnicity, maternal factors (e.g., BMI, education, and smoking during pregnancy), socioeconomic status, family income, and physical activity levels.

### Findings on association between dietary intake trajectories and health outcomes

A summary of results from 16 studies reporting associations between dietary intake trajectories from childhood or adolescence and health outcomes is presented in Table 2.

**TABLE 2:** Summary of studies reporting associations between dietary intake trajectories from early life and health outcomes.

	Dental caries	Adiposity outcomes	Cardiometabolic markers	Neurocognitive outcomes	Adverse liver outcomes	Gut microbiota
Food group intakes						
Core foods		Manohar et al., 2021 [9] (NS)				
Discretionary foods		Manohar et al., 2021 [9] (+)				
Sugary foods/drinks	Manohar et al., 2021 [9] (NS) Echeverria et al., 2022 [36] (+)					
Energy and nutrient intakes						
Energy				Toh et al., 2023 [40] (NS)	Anderson, 2015 [32] (+)	Oluwagbemigun et al., 2021 [30] (NS)
Carbohydrate				Toh et al., 2023 [40] (+ and –)	Anderson, 2015[32] (+ and NS)	Oluwagbemigun et al., 2021 [30] (+ and –)
Protein		Wright et al., 2017 [33] (+ and –)		Toh et al., 2023 [40] (+)	Anderson, 2015[32] (+ and NS)	Oluwagbemigun et al., 2021 [30] (NS)
Fat				Toh et al., 2023 [40] (+ and –)	Anderson, 2015[32] (+ and NS)	Oluwagbemigun et al., 2021 [30] (NS)
Fat subtypes					Anderson, 2015[32] (+ and NS)	
Fiber		Thorsteinsdottir, et al., 2023 [39] (NS)		Toh et al., 2023 [40] (+)		Oluwagbemigun et al., 2021 [30] (NS)
Starch					Anderson, 2015[32] (+ and NS)	
Free sugar	Ha et al., 2023 [37] (+)				Anderson, 2015[32] (+ and NS)	
Dietary pattern						
Healthy or guidelines	Hu et al., 2019 [34] (–)		Wu et al., 2021 [35] (NS)	Smithers, 2013 [31] (+)		
Predominantly breastmilk	Hu et al., 2019 [34] (NS)					
Easy-to-prepare foods	Hu et al., 2019 [34] (NS)					
Noodles and soup	Hu et al., 2019 [34] (NS)					
Traditional			Wu et al., 2021 [35] (NS)	Smithers, 2013 [31] (–)		
Vegetable and dairy			Wu et al., 2021 [35] (NS)			
High-carbohydrate foods			Wu et al., 2021 [35] (NS)			
Red meat			Wu et al., 2021 [35] (+)			
Discretionary foods				Smithers, 2013 [31] (–)		
Ready to eat				Smithers, 2013 [31] (NS)		
Diet quality score						
Healthy or high		Park et al., 2023 [38] (NS)∗	Kerr et al., 2018 [7] (NS)			
Moderately healthy			Kerr et al., 2018 [7] (NS)			
Moderately unhealthy			Kerr et al., 2018 [7] (NS)			
Unhealthy or low		Dalrymple et al., 2022 [28] (+) Park et al., 2023 [38](NS)∗	Kerr et al., 2018 [7] (+)			
Never healthy			Kerr et al., 2021 [8] (± and NS)			
Becoming less healthy			Kerr et al., 2021 [8] (+)			
Becoming healthy			Cosier et al., 2023 [6] (–)			
Always healthy			Cosier et al., 2023 [6] (–)			

(+): significant positive association (*P* < 0.05); (–): significant negative association (*P* < 0.05); NS, nonsignificant association (*P* ≥ 0.05). Park et al. [33] assessed breakfast quality.

#### Dental health in early childhood

Three of 4 studies reported significant associations between dietary intake trajectories (free sugar intake, intakes of food and drinks with added sugar, and dietary pattern) and dental outcomes in early childhood [34,36,37]. Ha et al. [37] found that children in the “high and increasing” compared with “low and decreasing” free sugar intake trajectory from ages 3 mo to 5 y had higher prevalence of dental caries at 5 y of age [prevalence ratio: 2.13; 95% CI: 1.23, 3.70]. Likewise, Echeverria et al. [36] found that “always intermediate," “increasing,” and “always high” sugar intake score trajectories (calculated as the sum of whether or not consuming various foods and drinks with added sugar) from ages 3 mo to 4 y was associated with a significantly higher prevalence of dental caries at 4 y than the “always low” sugar intake. Hu et al. [34] revealed that children following the high “guideline” dietary pattern (characterized by intakes of core food groups including fruits, vegetables, grains, dairy and lean meats), but not “predominately breastmilk,” “easy-to-prepare” or “noodles and soup” at ages 6 mo and from 6 to 12 mo had a lower prevalence of decayed surfaces among children at age 2–3 y (incidence risk ratio: 0.26; 95% CI: 0.12, 0.53). The remaining study found no evidence of an association between trajectories of sugary food and drink intake frequencies from ages 4 mo to 3 y and dental caries at age 3–4 y [9].

#### Adiposity outcomes in childhood and early adulthood

Of 5 studies reporting the associations between childhood dietary intake trajectories (intakes of protein or dietary fiber, core and discretionary food intakes, diet quality, breakfast quality) and adiposity outcomes in childhood [9,28,33,38,39], 3 studies revealed significant associations [9,28,33]. In a Filipino cohort, “usually high” protein intake (relative to recommendation) trajectory group from ages 2 to 22 y had lower BMI, lean, and fat mass at age 22 y than “normal” protein intake trajectory group [33]. When associations between protein intake across different ages (2, 11, 15, 19, and 22 y) and body composition at age 22 y were assessed, higher protein intake at age 2 y was associated with higher BMI and lean mass in females, but higher protein intakes at ages 11–22 y were associated with lower BMI, lean mass and fat mass at age 22 y in both males and females [33]. One Australian study observed that children with the “high” intake trajectory of discretionary foods(e.g., hot chips, potato crisps, sugary foods, and drinks), but not core food group intake trajectories, from ages 4 mo to 3 y had higher overweight risk at ages 3–4 y [odds ratio: 2.51; 95% CI: 1.16, 5.42] [9]. One study identified 5 diet quality trajectory groups/classes (“poor,” “poor-medium,” “medium,” “medium-better,” “best”) from 6 mo to 9 y of age. It found that a 1-class decrease (i.e., worsening) in diet quality trajectory was associated with higher percentage of body fat in SD units (β: 0.08 SD; 95% CI: 0.01, 0.15), higher BMI *z*-score (β: 0.08 SD; 95% CI: 0.00, 0.16), and lower percentage of lean mass (β: –0.08 SD; 95% CI: –0.14, –0.01) at 8–9 y of age [28]. With respect to studies that assessed dietary fiber intake trajectories (“low,” “moderate,” “high,” “unstable”) from ages 9 mo to 5 y [39] or breakfast quality trajectories (“high,” “low”) from ages 1.5 to 5 y [38], no evidence of significant association was found with BMI *z*-scores or overweight status at age 5 y in Australian children.

#### Cardiometabolic health in adolescence and adulthood

Four studies identified significant associations between dietary quality or pattern trajectories from early childhood (age <5 y) and cardiometabolic health outcomes in adolescence or adulthood up to age 41 y [[6], [7], [8],^35^]. Of these, 3 studies used data from the Longitudinal Study of Australian Children (LSAC) cohort. One of the 3 LSAC studies found children who followed “never healthy” compared with “always healthy” dietary quality score trajectory groups from ages 2 to 12 y had adverse cardiometabolic outcomes including higher resting heart rate (β: 2.6; 95% CI: 0.4, 4.7), a higher metabolic risk score (β: 0.23; 95% CI: 0.01, 0.45), lower arterial elasticity (β: –0.3; 95% CI: –0.6, –0.1), and lower carotid distensibility (β: –1.2; 95% CI: –1.9, –0.5) at ages 11–12 y [8]. Similarly, 2 other LSAC studies reported that children who followed the “never healthy” diet quality trajectory group from ages 4 to 11 y or 4 to 15 y had higher systolic or diastolic blood pressure at age 10–11 y [6] or higher resting heart rate at age 15 y [7] than children from the “always healthy” trajectory group. No differences in cardiometabolic outcomes were found among children from the “moderately healthy,” “becoming less healthy,” and “always healthy” trajectory groups [[6], [7], [8]]. In the remaining study, Wu et al. [35] reported that trajectories of “increased” or “stably medium” red meat dietary patterns, characterized by high intakes of red meat, pork, and sausages from ages 3–18 y to age 41 y were associated with a higher incidence of impaired fasting glucose at age 41 y (relative risk: 1.46; 95% CI: 1.12, 1.90) in Finish population. No association was found between other dietary pattern trajectory groups (“traditional Finnish,” “high carbohydrate,” “vegetable and dairy products,” “traditional Finish high carbohydrate,” and “healthy”) and impaired fasting glucose [35].

#### Other health outcomes in childhood and adolescence

Four studies found significant associations between childhood dietary intake trajectories (i.e., energy/macronutrients or dietary pattern) and other health outcomes including neurocognitive development [31,40], liver health outcomes [32], and gut microbiota [30]. Specifically, Toh et al. [40] found that macronutrient intakes, but not total energy intake, at age 6 mo and rate of change in intakes from ages 6 to 12 mo were associated with neurodevelopmental outcomes at age 24 mo, but not at age 54 mo. Significant positive associations were found between higher protein or dietary fiber intake and higher fine motor scores, but mixed findings were revealed for the association between carbohydrate intake and fine or gross motor or receptive language scores as well as for the association between fat intake and expressive or receptive language scores or fine motor scores [40]. Smithers et al. [31] found that the rate of change in “healthy” dietary pattern trajectory from ages 6 to 24 mo was associated with higher intelligence quotient (IQ) at age 8 y (β: 1.07; 95% CI: 0.17, 1.97), but not at age 15 y. In contrast, rate of change in “discretionary” (β: –0.73; 95% CI: –1.33, –0.14) and “traditional” (β: –0.41; 95% CI: –0.77, –0.04) dietary pattern from ages 6 to 24 mo was associated with lower IQ at age 15 y but not age 8 y [31]. No evidence of an association with IQ was found for the “ready to eat” dietary pattern. Anderson et al. [32] demonstrated that daily energy intakes at age 3, 7, and 13 y were associated with a higher likelihood of having more liver fat and adverse liver health markers at age 18 y. Inconsistent associations were found between macronutrient intakes and liver health outcomes, and the significant associations attenuated after adjustment for total energy intake [32]. Oluwagbemigun et al. [30] found a significant association of carbohydrate intake at age 1 y and rate of change in carbohydrate intake from ages 1 to 18 y, but not energy, protein, fat, or dietary fiber intakes, with gut microbiota composition (abundance of *Phascolarctobacterium, Dialister,* and *Desulfovibri)* at age 18 y.

### Risk of bias assessment

Of 16 studies, 2 studies were rated as high quality [38,39]. These studies met most of criteria [38,39], including having a clearly defined research question, being free of outcome bias at baseline, maintaining a drop-out rate <20%, providing comparisons between participants and dropouts, clearly defining outcomes, using reliable methods to assess exposure, ensuring the validity of outcome measures, employing repeated exposure measurements, making adequate adjustments for confounding factors, providing CIs, justifying sample size or describing power calculations, and declaring sources of funding. Most studies (*n =* 12) were rated as acceptable quality [[6], [7], [8], [9],^28^,[30], [31], [32], [33], [34], [35],^40^] that met most criteria above with some limitations in reporting or methodology. However, 2 remaining studies [36,37] were considered low quality, which involved inadequate adjustment for confounders, lack of comparison between participants and dropouts, and unreliable exposure or outcome assessment. Notably, there are large variations in the types of dietary assessment methods used across studies. About two-thirds of studies utilized 1–2 d food diaries/recalls, short dietary questions, unvalidated FFQ, or employed different dietary assessment methods across time points, providing less robust dietary intake data to assess longitudinal dietary trajectories than those that used multiple (≥3 d) 24h recalls, food records/diaries, or validated FFQ and consistent methods over time. Nevertheless, the quality of the studies did not appear to influence whether a study found a significant association (Table 3).

**TABLE 3:** Quality assessment of studies assessing the association between dietary intake trajectories from early life and health outcomes.

Reference (author, year)	Clearly focused question	Free of outcome at baseline	Drop-out rate (<20%)	Comparison between participants and dropouts	Clearly defined outcome	Assessment of exposure reliable	Validity of outcome measure	Repeated exposure measure	Adequate adjustment for confounding	CI provided	Sample size justification or power description	Declare of funding	Quality rating
Smithers et al., 2013 [31]	Y	N/A	N	Y	Y	N/C	Y	Y	Y	Y	N	Y	Acceptable
Anderson et al., 2015 [32]	Y	Y	N	N	Y	N	Y	Y	Y	Y	Y	Y	Acceptable
Wright et al., 2017 [33]	Y	N/A	Y	N	Y	N	Y	Y	Y	Y	N	Y	Acceptable
Kerr et al., 2018 [7]	Y	N/A	N	N	Y	Y	Y	Y	Y	Y	N	Y	Acceptable
Hu et al., 2019 [34]	Y	N	Y	N	Y	Y	Y	Y	N/C	Y	N	Y	Acceptable
Kerr et al., 2021 [8]	Y	N/A	N	N	Y	N/C	Y	Y	Y	Y	N	Y	Acceptable
Manohar et al., 2021 [21]	Y	N	N	N	Y	N/C	Y	Y	N/C	Y	N	Y	Acceptable
Oluwagbemigun et al., 2021 [30]	Y	Y	N/C	N	Y	Y	Y	Y	N/C	N	Y	Y	Acceptable
Wu et al., 2021 [35]	Y	N/A	N	N	Y	N	Y	Y	Y	Y	N	Y	Acceptable
Dalrymple et al., 2022 [28]	Y	N	N	Y	Y	Y	N/C	Y	Y	Y	N	Y	Acceptable
Echeverria et al., 2022 [36]	Y	N/C	Y	N	Y	N/C	Y	N/C	Y	Y	N	Y	Low
Cosier et al., 2023 [6]	Y	N/A	N	Y	Y	N/C	N/C	Y	Y	Y	N	Y	Acceptable
Ha et al., 2023 [37]	Y	N/C	N	N	Y	N	Y	N	Y	Y	N	Y	Low
Park et al., 2023 [38]	Y	N	N	Y	Y	Y	Y	Y	Y	Y	Y	Y	High
Thorsteinsdottir et al., 2023 [39]	Y	N	Y	Y	Y	Y	Y	Y	Y	Y	N	Y	High
Toh et al., 2023 [40]	Y	N/A	N	N	Y	N	Y	Y	Y	Y	Y	Y	Acceptable

Abbreviations: CI, confidence interval; N, no; N/A, not associated; N/C, not clear; Y, yes..

## Discussion

The present review provides longitudinal evidence demonstrating the vital contribution of changes in individual dietary components and whole diets from early life in the development of various health outcomes. It supports findings from past reviews that reported the prospective associations between dietary factors at 1 time point and later health outcomes [[41], [42], [43], [44], [45], [46]]. Most of the included studies examined the associations between trajectories of diet quality or patterns and health outcomes, with a few reporting on intake trajectories of macronutrients or food groups and associated health outcomes. Specifically, trajectories of dietary quality or pattern from infancy or early childhood were significantly associated with various health outcomes: dental caries and neurocognitive outcomes in early childhood, adiposity outcomes in childhood, and cardiometabolic markers in adolescence/ adulthood. Significant associations were also reported between macronutrient intake trajectories from infancy and adiposity, neurocognitive outcomes, liver outcomes, and gut microbiota. Intake trajectories of sugary or discretionary foods or drinks were associated with dental caries and adiposity, respectively.

The current review found an elevated risk of dental caries in children following trajectories of high free sugar intake or intake of sugary foods or beverages from infancy to early childhood [36,37]. The detrimental impact of high sugar intake on dental health is well established and could be attributable to several pathways, such as via promoting oral bacterial growth and plaque accumulation, and disrupting oral pH balance [42]. Our review findings further support the WHO guidelines to limit sugar intake for the prevention of dental caries. Relative to sugar intake, whether a healthy dietary pattern protects against dental outcomes remains unclear. It is encouraging that 1 study found a healthy dietary pattern trajectory during infancy was associated with a lower risk of dental caries in early childhood [34]. It is likely that a healthy dietary pattern contains essential nutrients such as calcium, vitamin D, and dietary fiber, which may support saliva production, healthy enamel, and tooth development [42]. Furthermore, children with a healthy dietary pattern might also adapt better oral health hygiene practices. Given existing research focused on dental outcomes in early childhood, more research will be desirable to understand the protective impact of dietary intake on longer-term dental outcomes.

Key dietary factors that showed significant longitudinal associations with adiposity outcomes in childhood or early adulthood included trajectories of protein intake, discretionary food intake, and unhealthy dietary quality from infancy or early childhood [9,28,33]. The associations between protein intakes with body weight outcomes differed by the life stage. Higher protein intake before age 2 y was linked with higher BMI and lean mass in early adulthood, whereas higher protein intake in adolescence or adulthood was linked to lower BMI, lean mass, and fat mass in early adulthood [33]. The detrimental effects of high protein intake in early life on body weight development are well-established and supported by the “early protein hypothesis” [47,48]. In contrast to the satiating effect of protein for body weight regulation in older children and adults, the “early protein hypothesis” suggests that excess protein intake may increase secretion of insulin growth factor-1 that could stimulate adipogenesis and gluconeogenesis and inhibit lipolysis, thus promoting susceptibility to body fat and weight gain in the long term [47,48]. For discretionary foods, the energy-dense and nutrient-poor nature could result in excess energy intake, positive energy balance, and, in turn, weight gain over the long term [44]. Similarly, poor diet quality is primarily characterized by low intake of core food groups and high intake of discretionary foods, which aligns with the observed inverse association between poorer diet quality and elevated body fat and BMI *z*-score, as well as reduced lean mass [28]. It should be noted that only 2 of the 5 studies examined body composition. Future research is warranted to examine the association between dietary intake trajectories and the long-term development of body composition (i.e., body fat and lean mass).

For cardiometabolic outcomes, an unhealthy or a low dietary quality trajectory in childhood or from childhood to adolescence was found to link with cardiometabolic markers such as resting heart rate, metabolic risk score, and blood pressure in adolescence and adulthood [[6], [7], [8]]. Furthermore, a significant association was revealed between the increasing and high dietary pattern trajectory of red meat, pork, and sausages from early childhood to adulthood and impaired fasting glucose in adulthood [35]. Apart from increasing obesity risk, low diet quality is characterized by high intakes of discretionary foods that are also high in saturated fat, sugar, and salt, which could contribute to a cascade of adverse interrelated metabolic responses such as inflammation, hypertension, insulin resistance, and dyslipidemia [44]. It has been widely recognized that red meat, pork, and sausages are rich sources of saturated fat, heme-iron, sodium, and nitrate, which can negatively affect cardiometabolic health [49]. Notably, cardiometabolic outcome measurements of the existing studies were captured in adolescence and early adulthood, which is not unexpected given the complexity of measurements. With the advancement in technology for assessing cardiometabolic risk measurements in younger children, emerging studies reported on cardioembolic outcomes in children [50]. There is potential for future studies to explore the relationship between diet and cardiometabolic risk in childhood.

Adequate nutrition from early life is vital for neurocognitive development in children. Our review identified evidence to support a weak association between dietary pattern trajectories from infancy to toddlerhood and IQ scores in childhood and/or adolescence, with the “healthy” dietary pattern associated with higher IQ scores and the “discretionary” and “traditional” dietary pattern linked with lower IQ scores [31]. Notably, the “healthy” dietary pattern is characterized by breastfeeding, intakes of fruits, vegetables, cheese, and herbs. In addition to the documented benefits of breastfeeding on cognitive development, this pattern may provide nutrients (e.g., iron, zinc, and B vitamins) that support cognitive development. Significant positive associations were also found between macronutrient intake trajectories (e.g., protein or dietary fiber) during infancy and language and fine/gross motor outcomes in early childhood, but findings for fat or carbohydrate intake trajectories were mixed [40]. The mechanisms underlying macronutrient intakes and neurocognitive development in children remain equivocal and require further investigation [51].

Significant associations were also found between “high energy” intake trajectory from early childhood to adolescence and adverse liver markers in early adulthood [32]. Intakes of specific macronutrients were not linked with liver markers [32]. Lastly, carbohydrate intake trajectory from infancy to early adulthood was associated with more positive gut microbiota composition in early adulthood [30]. However, how energy and carbohydrate intake influence liver markers and gut microbiota, respectively, remains to be better understood.

No evidence of an association was reported between breakfast quality or dietary fiber intake trajectories and adiposity outcomes from infancy to early childhood in either of the 2 studies involving secondary data analysis of a randomized controlled trial with follow-up of children until age of 5 y [38,39]. The small sample size of the studies may have limited the power to detect significant associations. Moreover, the trial involved a highly educated cohort, which may have led to more homogenous dietary intake patterns and a lower prevalence of obesity compared with populations with diverse educational backgrounds. This relative homogeneity likely reduced data variability, thereby diminishing the ability to detect significant associations between dietary intake and obesity outcomes. Notably, 2 studies assessed the associations of dietary pattern or macronutrient intake trajectory from infancy with neurocognitive outcomes at 2 subsequent time points in childhood or adolescence and found a stronger association at the earlier time points [31,40]. These findings suggest that the impact of early dietary intake may diminish over time, or other environmental influences may come into play as children grow older. Also, the variations in assessment of the outcomes or decreased sample size with longer follow-up may also explain the weakening of the associations.

Our review has several strengths. To our knowledge, the current review is the first to systematically review and synthesize the longitudinal evidence on the associations between dietary intake trajectories from early life and concurrent and subsequent health outcomes. The inclusion of studies that used various trajectory modeling approaches (multilevel mixed effect models, GBTM, LCTM) to assess longitudinal changes in dietary intake over 3 or more time points and their long-term health effects is a key strength. The multilevel mixed effect models estimate the average trajectory of diet over time within a population, enabling the identification of key time points of dietary change and when the association with health outcome emerged or how the association evolved over time. In contrast, GBTM identifies heterogeneous groups within a population that follow distinct dietary trajectories. LCTM, a type of growth mixture modeling, allows further individual-level variability within each dietary trajectory group. From which, differential associations between dietary trajectory groups and health outcomes can be examined. Studying the longitudinal trajectories of diet and associations with health outcomes reveals critical information on when and who to target for dietary intervention. Another strength of the current review is that two-thirds of the included studies are of high or acceptable study quality, further supporting the robustness of the findings.

Limitations of the current review should also be acknowledged. Due to the observational nature of the included studies, the current systematic review cannot establish causal relationships between dietary intake trajectories and health outcomes, and unmeasured or residual confounding is possible. Dietary intake is self-reported, and reporting bias cannot be dismissed. The quality of dietary assessment was low in two-thirds of the included studies, undermining the overall quality of dietary data. Also, several studies explored health outcomes at the last time point of dietary intake trajectories, which implies that the directionality of associations at the last time point often remains unclear. Nevertheless, the current review provides valuable insights into the temporal order of the relationship between dietary intake and health outcomes. The bulk of the literature explored the association between dietary quality or pattern, or macronutrient trajectories, and health outcomes, and only 2 studies assessed food group intake trajectories. To comprehensively examine the influences of diets from the reductionist and holistic approaches, it would be desirable for further research to assess the longitudinal associations between different aspects of diet (e.g., food groups and micronutrient intake trajectories) and health outcomes. Furthermore, most included studies assessed health outcomes from childhood to adolescence, with only 4 studies examining health outcomes in adulthood from ages 18 to 41 y. The role of dietary intake from childhood or adolescence on long-term health outcomes in adulthood remains to be further elucidated. Associations between various dietary intakes and health outcomes were assessed. However, the number of available studies for each health outcome remained limited. Previous reviews showed that emerging studies explored factors associated with trajectories of specific nutrients or food groups, such as intakes of vitamin D, fruits and vegetables, sugar-sweetened beverages, and fast foods, but their association with health outcomes was not assessed [16]. These studies and others can be extended to include measurements of health outcomes. Moreover, given the heterogeneity in dietary intake trajectories being examined, a meta-analysis could not be conducted to quantitatively synthesize the findings. Lastly, the inclusion of only English-language papers may have restricted the review's scope, potentially excluding relevant studies in other languages.

Our review has important public health implications and will grant valuable insights into life course nutritional epidemiology literature. In addition to defining key dietary factors that showed enduring impact on health outcomes, the present review also highlights infancy and early childhood as a critical window when longitudinal associations between dietary intake and health outcomes started to emerge. Various studies assessing trajectories of diet quality or dietary pattern from infancy or early childhood identified stable trajectories of diet quality from infancy or early childhood to later childhood [6,28] as well as consistent dietary patterns from infancy to toddlerhood [31,40]. Stable trajectories of sugar or fiber intake trajectories from infancy to early childhood were also reported [36,39]. Trajectories of diet quality, dietary pattern, discretionary food intake, energy or macronutrient intakes from infancy or early childhood were found to play a role in various health outcomes in early childhood, adolescence, or adulthood. This finding corroborates the Developmental Origins of Health and Disease theory, which highlights the importance of infancy and early life in programming of later health and disease [52]. These findings suggest that healthy diets should start in infancy or early childhood and focus on promoting a healthy dietary pattern or diet quality (high in core food groups and low in discretionary foods, sugar, or red meat). Early intervention to promote healthy diets will facilitate the prevention of dental caries and promote optimal neurocognitive development in childhood as well as the reduction of obesity and cardiometabolic risk in adolescence or early adulthood. Given the limited number of studies that assessed energy or macronutrient intake trajectories from early life and neurocognitive, liver makers, or gut microbiota in early childhood, further longitudinal research is needed to consolidate these findings. Also, more longitudinal studies with longer duration of follow-up are needed to understand the associations between trajectories of specific nutrients or food groups and long-term health outcomes in adulthood. Such information will contribute high-quality longitudinal evidence to facilitate the refinement of dietary guidelines, such as nutrient reference values and food-based dietary recommendations.

In conclusion, the present systematic review demonstrates that dietary intake trajectories (e.g., energy, macronutrients or sugar intakes, sugary or discretionary foods, dietary quality, and dietary pattern) from infancy or early childhood were associated with various health outcomes such as dental caries, adiposity, cardiometabolic markers, neurocognitive development, liver function, or gut microbiota from early childhood up to adulthood. These findings highlight infancy and early childhood as a critical window when the impact of diet on health starts to emerge and provide new longitudinal evidence to reinforce the importance of establishing healthy dietary habits from early life for long-term health promotion and chronic disease prevention. However, further research with longer duration of follow-up into adulthood is warranted to explore trajectories of dietary intake, particularly specific food groups and nutrients, and its long-term impact on health outcomes.

## Author contributions

The authors’ responsibilities were as follows – MZ: conceived and designed the study; MZ, SYP: conducted literature search and wrote the initial draft; SYP, KAB, MZ: conducted study selection, data extraction, and study quality assessment; MF-FC, SG, BX: assisted with results interpretation; and all authors: critically reviewed and approved the final version of the manuscript and agreed to be accountable for all aspects of the work.

## Data availability

Data described in the manuscript, codebook, and analytic code will be made available on request pending application and approval.

## Funding

MZ is supported by an Australian Research Council Discovery Early Career Researcher Award (DE240100635).

## Conflict of interest

MZ reports financial support was provided by Australian Research Council. All other authors report no conflicts of interest..
