# Supplementary material for: Dietary Intake Trajectories from Early Life and Associated Health Outcomes: A Systematic Review
Source: Adv Nutr. 2025 Sep 29;16(11):100528. doi: 10.1016/j.advnut.2025.100528 (PMC12590264; doi:10.1016/j.advnut.2025.100528)
Supplement: multimedia component 1 [file mmc1.docx]

**Supplementary table 1. Summary of reported dietary variable and health outcome definition**

| **Author, year** | **Sample** | **Dietary variable** | **Dietary variable**  **Definition** | **Health outcomes** | **Specific outcome** |
| --- | --- | --- | --- | --- | --- |
| Smithers, 2013 | UK, ALSPAC | Dietary pattern | **Healthy:**  Breast feeding at 6mo, raw fruit, vegetables, cheese  and herbs  **Discretionary:**  biscuits, chocolates, etc  **Traditional:**  meat, cooked vegetables,  puddings, etc  **Ready-to-eat:**  bread, cereals, etc | Neurocognitive outcomes  (Measured) | Intelligence quotient (IQ) |
| Anderson, 2015 | UK,  ALSPAC | Energy and nutrient intakes | Energy, total  carbohydrate, sugar,  starch, total fat,  monounsaturated  fat, poly unsaturated  fat, saturated fat and  total protein | Adverse Liver outcomes (Measured) | Liver fat and  stiffness,  blood liver  markers |
| Wright, 2017 | Philippines, CLHNS study | Protein intakes | Protein intakes relative to needs (g/kg body weight) | Overweight and obesity (Measured) | Body Mass Index (BMI), lean mass, and fat mass |
| Kerr, 2018 | Australia, PEAS study | Diet quality score | 0 (lowest)-14 (highest) points:  fruit, vegetables, water, milk products /alternatives, fatty foods, sugary foods, sweetened drinks | Cardiometabolic markers (Measured) | Blood  pressure, resting  heart rate, pulse  wave velocity,  carotid intima  media thickness,  retinal arteriole-  to-venule ration) |
| Hu, 2019 | Singapore, GUSTO study | Dietary pattern | **Predominantly breastmilk**  **Guidelines**:  rice porridge, fish, meat, fresh fruit, and vegetables  **Easy-to-prepare foods**  **Noodles (in soup) and seafood** | Dental caries (Measured) | Dental caries present/absent, decayed teeth, decayed surfaces |
| Kerr, 2021 | Australia, LSAC | Diet quality score | 0 (lowest) -14 (highest) points:  fruit, vegetables, water, milk products /alternatives, fatty foods, sugary foods, sweetened drinks | Cardiometabolic markers-  Structural phenotypes, Metabolic risk score  (Measured) | Cardiovascular phenotypes: resting heart rate, blood pressure, pulse wave velocity, carotid elasticity/distensibility,  Structural phenotypes: carotid intima-media thickness, retinal microvasculature,  Metabolic risk score: BMI z-score, systolic blood pressure, high-density lipoproteins cholesterol, triglycerides, and glucose (Measured) |
| Manohar, 2021 | Australia, HSHK study | Food groups | **Intake frequency**  **Core Foods:**  dairy, grains, fruits, vegetables, and meat/alternatives  **Discretionary Foods:**  added fats/salt/sugars  **Sugary Foods:**  sugar containing items | Overweight/  obesity and dental caries (Measured) |  |
| Oluwagbemigun, 2021 | Germany, DONALD study | Energy and macronutrient intakes | Energy, carbohydrate, fibre, protein, and fat | Gut microbiota composition (Measured) | *Phascolarctobacterium, Dialister, Desulfovibrio, etc* |
| Wu, 2021 | Finland, YFS study | Dietary pattern | **Traditional Finnish**:  rye, potatoes, butter, milk, coffee, sausages, low fruit/berries  **High carbohydrate foods**:  wheat, margarine, oils, sugar, milk, beef, eggs  **Vegetable and dairy products:**  vegetables, fruits, cheese, other dairy products, tea, beef, alcoholic beverages  **Traditional Finnish and high** carbohydrate:  Wheat, other grains, rye, potatoes, butter, sausages, sugar  **Red meat:**  pork, other meats, sausages  **Healthy:**  vegetables, legumes, nuts, fruits, fish, cheese, dairy products, tea, lean meats, eggs | Cardiometabolic markers - Impaired fasting glucose (IFG) (Measued) |  |
| Dalrymple, 2022 | UK, SWS | Diet quality score | 49 foods groups –  High intakes of fruit and vegetables, wholemeal bread, rice and pasta, yogurt and breakfast cereals, and Low intakes of chips & roast potatoes, sugar, white bread, red and processed meat, full-fat dairy products, crisps, Yorkshire pudding & savoury pancakes, sweets & chocolate, tea & coffee, tinned vegetables, cakes & biscuits, and soft drinks  Scores calculated using principal component coefficients | Overweight/  obesity (Measured) | BMI z-score, child overweight status |
| Echeverria, 2022 | Brazil, 2015 Pelotas Birth Cohort | Food group | Sugar consumption scores were calculated as the sum of all sugar containing foods and drinks (consumer vs non-consumer) | Dental caries (Measured) | Dental caries experience/ Cavitated dental caries |
| Cosier, 2023 | Australia, LSAC | Diet quality score | 0 (lowest) -14 (highest) points:  fruit, vegetables, water, milk products /alternatives, fatty foods, sugary foods, sweetened drinks | Cardiometabolic markers (Measured) | Blood pressure: Systolic Blood Pressure (SBP), Diastolic Blood Pressure (DBP) |
| Ha, 2023 | Australia, SMILE study | Energy and macronutrient intakes | Free sugar intakes in grams | Dental caries (Measured) |  |
| Park, 2023 | Australia, INFANT study | Diet quality score  (Breakfast quality index (BQI)) | 0 (lowest)-9 (highest) points:  cereals, wholegrains, dairy products, fruit, vegetables, calcium, energy, absence of added sugar, absence of butter/margarine | Overweight/obesity (Measured) | BMI z-score, child overweight status |
| Thorsteinsdottir, 2023 | Australia, INFANT study | Energy and macronutrient intakes | Fibre intakes | Overweight/obesity  (Measured) | BMI z-score, child overweight status |
| Toh, 2023 | Singapore, GUSTO study | Energy and macronutrient intakes: | Energy, protein, total fat, carbohydrate, dietary fibre | Neurocognitive outcomes  (Measured) | BSID-III test: cognition, expressive language, receptive language, fine motor and gross motor, KBIT-2 test: verbal, non-verbal IQ subtest) |
